# New Insights on Old Biomarkers Involved in Tumor Microenvironment Changes and Their Diagnostic Relevance in Non-Small Cell Lung Carcinoma

**DOI:** 10.3390/biom11081208

**Published:** 2021-08-13

**Authors:** Katarzyna Wadowska, Piotr Błasiak, Adam Rzechonek, Iwona Bil-Lula, Mariola Śliwińska-Mossoń

**Affiliations:** 1 Department of Medical Laboratory Diagnostics, Division of Clinical Chemistry and Laboratory Haematology, Wroclaw Medical University, Borowska 211A, 50-556 Wroclaw, Poland; iwona.bil-lula@umed.wroc.pl (I.B.-L.); mariola.sliwinska-mosson@umed.wroc.pl (M.Ś.-M.); 2 Department and Clinic of Thoracic Surgery, Wroclaw Medical University, Grabiszyńska 105, 53-439 Wroclaw, Poland; piotr.blasiak@umed.wroc.pl (P.B.); adam.rzechonek@umed.wroc.pl (A.R.); 3 Department of Thoracic Surgery, Lower Silesian Center for Lung Diseases, Grabiszyńska 105, 53-439 Wroclaw, Poland

**Keywords:** lung cancer, non-small cell lung carcinoma, adenocarcinoma of lung, squamous cell carcinoma of lung, tumor microenvironment, malignant progression, biochemical tumor markers, diagnostic biomarkers, differential diagnoses, ROC analyses

## Abstract

Background: Lung cancer is a multifactorial disease with a heterogeneous tumor group that hampers diagnostic and therapeutic approaches, as well as understanding of the processes that underlie its pathogenesis. Current research efforts are focused on examining alterations in the tumor microenvironment, which may affect the pathogenesis and further malignant progression in lung cancer. The aim of this study was to investigate changes in the levels of biomarkers involved in the lung tumor microenvironment and their diagnostic utility in differentiating lung cancer subtypes and stages. Methods: This study comprised 112 lung cancer patients, 50 with adenocarcinoma, 35 with squamous cell carcinoma, 13 with other non-small cell lung carcinoma subtypes, and 14 with other lung neoplasms than non-small cell lung carcinoma. Tumor markers (CEA, CYFRA 21-1, and NSE) were measured in the patients’ sera and plasmas, along with IL-6, TNF-α, SAA_1_, CRP, MMP-2, MMP-9, glucose, lactate, and LDH, utilizing enzyme-linked immunosorbent assays, enzyme immunoassays, and automated clinical chemistry and turbidimetry systems. The results were statistically analyzed across patient groups based on the subtype and stage of lung cancer. Results: Glucose concentrations showed statistically significant (*p* < 0.05) differences both between lung cancer subtypes and stages, with the highest levels in patients with other lung neoplasms (me = 130.5 mg/dL) and in patients with stage IIB lung cancer (me = 132.0 mg/dL). In patients with advanced lung cancer, IL-6 and LDH had considerably higher concentration and activity. There was also a significant positive correlation between IL-6 and MMP-9 in adenocarcinoma and SqCC, with correlation coefficients of 0.53 and 0.49, respectively. The ROC analyses showed that the best single biomarkers for distinguishing adenocarcinoma from squamous cell carcinoma are glucose, CRP, and CYFRA 21-1; however, their combination did not significantly improve sensitivity, specificity, and the AUC value. The combinations of IL-6, glucose, LDH and CEA, IL-6, SAA1, MMP-9, and lactate can distinguish patients with stage IIB lung cancer from those with stage IIA with 100% sensitivity, 100% specificity, and with an AUC value of 0.8333 and 1.0000, respectively, whereas the combination of CEA, IL-6, and LDH can identify patients with stage IIIA lung cancer from those with stage IIB with 72.73% sensitivity, 94.44% specificity, and an AUC value of 0.8686. Conclusion: There is a link between biomarkers of tumor microenvironment changes and tumor markers, and combinations of these markers may be clinically useful in the differential diagnosis of adenocarcinoma and squamous cell carcinoma, as well as lung cancer stages IIB and IIA, and IIIA and IIB.

## 1. Introduction

Cancer is one of the multifactorial diseases that is thought to be caused by complex interactions between genetic and environmental factors. The precise pathogenesis of lung cancer has not yet been fully understood [1,2,3]. Based on current knowledge, lung neoplasm results from the final stage of multi-stage bronchial cells carcinogenesis, with progressively increasing genetic and epigenetic changes due to exposure to environmental factors and patients’ individual predispositions to lung cancer [4,5]. Recent studies on gene and protein expression focused on early diagnosis of the type and stage of lung cancer in relevance to treatment options, simultaneously providing information on the initiation, progression of tumorigenesis, and differences between subtypes [6]. Growing evidence shows that lung cancer represents a group of histologically, cellularly, and molecularly heterogeneous tumors within the same histological type, which influences response and resistance to targeted therapies as well as diagnostic processes [7].

Non-small cell lung carcinoma (NSCLC) is the most common type of lung cancer, accounting for up to 85% of cases. The tissue composition and molecular landscape of NSCLCs are both heterogeneous, affecting clinical decision making in lung cancer treatment. NSCLC must be divided into two types: adenocarcinoma and squamous cell carcinoma (SqCC), each of which has a unique set of clinically actionable mutations [8,9,10,11,12]. Lung tumor cells with acquired somatic mutations influence cytokine and chemokine secretion, modifying the chemoattractant properties of the tumor microenvironment (TME). Changes in the TME, remodeling of the extracellular matrix (ECM), pro- and anti-inflammatory processes, and cell metabolism are affected by neoplastic progression, but they also affect further progression and metastasis of the tumor. These changes can be examined by evaluating particular inflammatory and metabolic markers, which, in turn, may be considered diagnostic biomarkers [13,14,15,16,17,18].

Diagnosis of lung cancer requires improvement. Most patients are diagnosed with locally advanced stage of pulmonary neoplasm or metastatic disease and have a 1-year survival rate of about only 20%. In addition, the 5-year survival rate of patients with lung cancer is approximately 15%, which in combination with the fact that lung cancer is the most prevalent cancer in the world, accounting for 2.09 million cases, makes it the most common cause of cancer death (1.76 million deaths in 2018) [19,20,21]. These data suggest an urgent need for early detection of lung neoplasm and to differentiate subtypes with available target therapies. However, early detection of neoplastic changes requires adequate diagnostic methods characterized by high sensitivity, specificity, and non-invasiveness [22].

**Figure 1:**
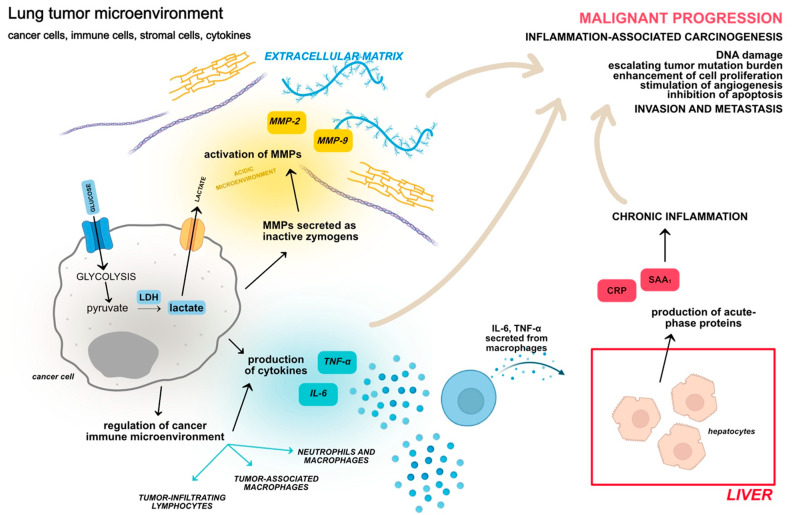
A simplified diagram depicting the role of glucose, LDH, lactate, MMP-2, MMP-9, TNF-α, IL-6, SAA1, and CRP in lung cancer progression. Created with BioRender.com and Affinity Designer. TME is a complex mixture of tumor cells, extracellular matrix, and an inflammatory microenvironment including immune cells and cytokines that plays an essential part in tumorigenesis and progression [14,17,27]. The unrestricted proliferation of cancer cells influences TME, manifesting itself, among other things, in increased glucose uptake. Glucose is glycolyzed after it enters the cell through the glucose transporters (GLUT). Glucose is converted to pyruvate during glycolysis, and this conversion provides the carbon needed to produce precursors for nucleotide, protein, and lipid synthesis in lung tumor cells. In subsequent steps, LDH reduces pyruvate to lactate under anaerobic conditions. Even in the presence of oxygen, lung cancer cells metabolize glucose via lactic acid fermentation, and lactate is secreted into the extracellular space by monocarboxylate transporters (MCT), causing the acidification of the microenvironment [28,29,30,31,32]. The acidic microenvironment activates MMPs, which are secreted as an inactive zymogens by tumor cells. MMPs are metastasis-associated proteins that alter cellular signals, regulate the expression of cytokines and growth factors, and cause the degradation of extracellular matrix and cellular membrane components, resulting in cancer cells invasion and metastasis. Lactate excretion also affects the function of immune cells and triggers immune escape of tumor cells. Immune cells within the TME are divided into tumor-antagonizing and tumor-promoting immune cells and regulated by the cancer cells. Effector T cells, natural killer (NK) cells, M1-polarized macrophages, and N1-polarized neutrophils secrete pro-inflammatory cytokines, chemokines, and reactive oxygen/nitrogen species to induce the cytotoxicity in cancer cells and recruit other cells with antitumor activity [33]. Many immune cells act through the secretion of TNF-α and IL-6, which have a synergistic relationship. TNF-α expression can cause a 60-fold increase in IL-6 production, while IL-6 is able to induce expression of TNF-α in NSCLC cells. These macrophage-secreted cytokines regulate liver cell production of the non-specific acute-phase proteins SAA_1_ and CRP, which are linked to inflammation [34,35,36,37,38,39,40]. Elevated IL-6 expression is associated to the transition from acute to chronic inflammation, which contributes to the development of inflammation-associated carcinogenesis. Chronic inflammatory mechanisms may contribute to tumor formation, growth, and metastasis via DNA damage, enhanced cell proliferation, angiogenesis stimulation, and apoptosis inhibition [41]. LDH—lactate dehydrogenase; MMP-2—matrix metalloproteinase-2; MMP-9—matrix metalloproteinase-9; TNF-α—tumor necrosis factor-α; IL-6—interleukin-6; CRP—C-reactive protein; SAA_1_—serum amyloid A_1_; TME—tumor microenvironment; GLUT—glucose transporter; MCT—monocarboxylate transporter; NK—natural killer.

Widely described cancer antigens, such as cytokeratin 19 fragment (CYFRA 21-1), carcinoembryonic antigen (CEA), and neuron-specific enolase (NSE), have confirmed usefulness as a tumor biomarker panel in the diagnosis of lung cancer, but they lack high sensitivity and specificity [4,23,24]. Combining tumor markers with serum proteins appears to be an approach that has the potential to increase the diagnostic value of tumor markers. The study of the relationship between interleukin-6 (IL-6) and tumor markers in breast and colorectal cancers is an example of such a combination, as well as one of the research hotspots [25,26]. We decided to take this study a step further and investigate the relationship between tumor markers with pro- and anti-inflammatory cytokines (IL-6, tumor necrosis factor-α—TNF-α), as well as matrix metalloproteinases-2 and -9 (MMP-2, MMP-9), both of which have been shown to be essential in the tumor microenvironment. Furthermore, we chose to look at easily accessible markers such as acute-phase proteins (C-reactive protein—CRP, serum amyloid A_1_—SAA_1_) that are linked to the expression of pro- and anti-inflammatory cytokines, as well as metabolic markers associated with the Warburg effect, i.e., glucose metabolism in cancer cells (glucose, lactate dehydrogenase—LDH, and lactate). Figure 1 shows the relationship between the chosen biomarkers and their roles in the tumor microenvironment as well as cancer progression.

The aim of this study was to see if there were any changes in the levels of evaluated biomarkers between lung cancer subtypes and stages. We decided to investigate the relationship between the assessed tumor and the inflammatory and metabolic markers to see if combining them could improve their diagnostic value in patients with lung adenocarcinoma and squamous cell carcinoma, as well as chosen lung cancer stages IIB and IIA, IIIA and IIB, resulting in better therapeutic decision making.

## 2. Materials and Methods

### 2.1. Patients

The research group consists of 112 consecutive patients recruited by the Department of Thoracic Surgery at the Lower Silesian Center for Lung Diseases in Wroclaw, Poland. Before collecting the blood samples, the experiment was approved by the local Ethics Committee at Wroclaw Medical University, and all patients signed written informed consent to participate in the study. Prior to any surgical treatment, venous blood samples were collected into tubes with ethylenediaminetetraacetic acid (EDTA) anticoagulant and tubes with clot activator from all patients. Blood samples were centrifuged at 2000× *g* for 8–10 min at room temperature to separate plasma and serum, which were then stored at −80 °C until use. The average storage time was less than 6 months.

The research group was surgically treated, either with a thoracotomy or with video-assisted thoracoscopic (VAT) surgery. The majority of the patients (61 out of 112, 54.46%) underwent lobectomy, with the remainder undergoing wedge resection (28 out of 112, 25.00%), biopsy (9 out of 112, 8.04%), segmentectomy (7 out of 112, 6.25%), bilobectomy (5 out of 112, 4.46%), and pulmonectomy (2 out of 112, 1.79%). A histopathological examination was performed on the tumor tissue obtained, which provided us with the necessary information about the diagnosis of lung cancer. The information about the studied group was completed by clinical and pathological data obtained from hospital medical reports using the Asseco Medical Management Solutions (AMMS) IT system. The characteristics of the studied group are shown in Table 1.

Based on the National Comprehensive Cancer Network (NCCN) Clinical Practice Guidelines in Oncology, we divided the study group by histopathological type into NSCLC and non-NSCLC patients (one with small cell lung carcinoma [SCLC], four with benign pulmonary nodules, two with mesothelioma, and seven with metastases of breast, colorectal, stomach, or esophageal cancer to the lungs), and then further subdivided the NSCLC group into adenocarcinoma = 50, SqCC = 35, and others (five patients with large cell neuroendocrine carcinoma, four patients with typical carcinoid, two with pleomorphic carcinoma, and two with not otherwise specified carcinoma). We also used the American Joint Committee on Cancer’s (AJCC) 8th TNM Staging System to divide the study group by lung cancer stage. These groups were compared among themselves in the statistical analyses.

### 2.2. Methods

Serum blood samples were used to measure levels of CEA, NSE, IL-6, TNF-α, SAA1, MMP-2, and MMP-9 by the enzyme-linked immunosorbent assay (ELISA). The assay was performed according to the manufacturer’s instruction (CEA ELISA/Catalog Number: EIA 1868, DRG Instrument GmbH, Marburg, Germany; Human Enolase 2/Neuron-specific Enolase Quantikine ELISA Kit/Catalog Number: DENL20, R&D Systems, Inc., Minnesota, MN, USA; Human IL-6 DuoSet ELISA/Catalog Number: DY206, R&D Systems, Inc., Minnesota, MN, USA; Human TNF-alpha DuoSet ELISA/Catalog Number: DY210, R&D Systems, Inc., Minnesota, MN, USA; Human Serum Amyloid A1 DuoSet ELISA/Catalog Number: DY3019-05, R&D Systems, Inc., Minnesota, MN, USA; Total MMP-2 Quantikine ELISA Kit/Catalog Number: MMP200, R&D Systems, Inc., Minnesota, MN, USA; and Human MMP-9 Quantikine ELISA Kit/Catalog Number: DMP900, R&D Systems, Inc., Minnesota, MN, USA). CEA was immobilized with monoclonal anti-CEA antibody and detected by monoclonal anti-CEA antibody conjugated to horseradish peroxidase (HRP). NSE/MMP-2/MMP-9 was immobilized with specific for human enolase 2/total MMP-2/human MMP-9 monoclonal antibody and was detected using polyclonal antibody specific for human enolase 2/total MMP-2/for human MMP-9 conjugated to HRP. IL-6/TNF-α/SAA_1_ was immobilized by mouse anti-human IL-6 capture antibody/mouse anti-human TNF-α capture antibody/mouse anti-human serum amyloid A1 capture antibody and was detected using biotinylated goat anti-human IL-6 detection antibody/biotinylated goat anti-human TNF-α detection antibody/biotinylated mouse anti-human serum amyloid A1 detection antibody along with streptavidin conjugated to HRP. In each assay, the reaction was developed using a tetramethylbenzidine (TMB) substrate solution. The substrate reaction was stopped, and the extinction was measured at 450 nm with the correction read at 540 nm using an ELISA reader. The kits provided standards ranging from 0 ng/mL to 100 ng/mL for CEA, 0 ng/mL to 20 ng/mL for NSE, 0 pg/mL to 600 pg/mL for IL-6, 0 pg/mL to 1000 pg/mL for TNF-α, 0 ng/mL to 100 ng/mL for SAA_1_, 0 ng/mL to 32 ng/mL for MMP-2, and 0 ng/mL to 20 ng/mL for MMP-9. The standard curve was used to calculate the concentrations of measured biomarkers and was linearized by plotting the logs of the standards’ concentrations versus the logs of the measured absorbances. If the measurements were out of the range, the samples were diluted, and the concentration read from the standard curve was multiplied by the dilution factor.

The concentrations of CYFRA 21-1 were determined using an enzyme immunoassay (EIA), which is a non-competitive immunoassay. The assay was carried out in accordance with the manufacturer’s instructions (CYFRA 21-1 EIA/Catalog Number: 211-10, Fujirebio Diagnostics AB, Göteborg, Sweden), with two monoclonal antibodies (Anti-CYFRA 21-1 monoclonal antibody from mouse and Biotin Anti-CYFRA 21-1 monoclonal antibody from mouse) directed against two separate antigenic determinants of soluble fragments of cytokeratin 19. The assay procedure was similar to that of ELISA, and the kit contained standards ranging from 0 ng/mL to 45.4 ng/mL. The methods for calculating the results remained the same.

Plasma samples were used to measure concentrations of CRP, glucose, lactate, and LDH. The assay was performed by commercial test kits according to the manufacturer’s instruction (C-REACTIVE PROTEIN/Catalog Number: 31321, BioSystems S.A., Barcelona, Spain; GLUCOSE-HK/Catalog Number: 11538, BioSystems S.A., Barcelona, Spain; LACTATE/Catalog Number: 11736, BioSystems S.A., Barcelona, Spain; and LACTATE DEHYDROGENASE, LDH/Catalog Number: 11580, BioSystems S.A., Barcelona, Spain) using BioSystems a15 analyzer.

### 2.3. Statistical Analysis

Prior to all statistical analyses, the estimated concentrations of the evaluated biomarkers were logarithmically transformed to achieve normal distribution. The Shapiro-Wilk test was used to determine if the data obtained for each biomarker had a normal distribution across all analyzed groups.

The analysis of variance (ANOVA) method was used to compare the concentrations of biomarkers in four groups of lung cancer patients (adenocarcinoma—ADC, squamous cell carcinoma—SqCC, other NSCLCs—Other, and not NSCLCs) as well as seven groups of lung cancer stage. Before choosing the ANOVA test for the analyses, the Brown–Forsythe test was used to verify the equality of the variables in groups. In the analysis, the F Welch’s test was performed. In the lack of a normal distribution for the biomarker, the Kruskal–Wallis one-way ANOVA on ranks was performed to compare the concentrations. After determining whether there are any differences in biomarker levels across groups, post hoc tests were conducted to determine which groups vary statistically significantly. Tukey’s honestly significant difference (HSD) test was used in the post hoc analysis.

The pairwise correlations were computed using Pearson correlation in variates to evaluate the associations between the levels of all analyzed proteins in lung cancer patients with adenocarcinoma, SqCC, other NSCLCs, other lung neoplasms than NSCLC groupings, and overall.

The final step of statistical analysis was to evaluate the diagnostic utility of our biomarkers in distinguishing adenocarcinoma and SqCC, as well as lung cancer stages IIB and IIA, and IIIA and IIB. The ability of the assessed biomarkers to differentiate chosen groups was estimated using logistic regression models. To begin, the generalized linear model (GLM) was used to exclude out the effect of patients’ age, gender, and smoking status on biomarker differences across groups. The sensitivity, specificity, and area under the curve (AUC) for each individual marker were calculated using the receiver operating characteristic (ROC) curve analysis. Following that, the generalized linear model with Akaike information criterion (AIC) estimation was employed to find the optimal model and the combination of biomarkers with the best diagnostic value. The ROC analysis was not performed for patients with other subtypes of NSCLC and other lung cancers than NSCLC due to the smaller sample size. In all these analyses, a *p*-value of 0.05 or less was considered statistically significant.

All statistical analyses were carried out using TIBCO Software Inc. (Palo Alto, CA, USA) (2017). Statistica (data analysis software system), version 13 (http://statistica.io, accessed on 10 May 2021 and 30 July 2021) with the additional Plus Package (version 5.0.96), was used.

## 3. Results

### 3.1. Patients’ Characteristic

Tumor (CEA, CYFRA 21-1, and NSE), inflammatory markers (IL-6, TNF-α, SAA_1_, CRP, MMP-2, and MMP-9), and metabolic markers (glucose, lactate, and LDH) were measured in the sera and plasmas of 112 lung cancer patients, who were classified into four groups based on subtype and seven groups based on stage. The research included 41 women (21 with adenocarcinoma, 8 with SqCC, 5 with other NSCLCs, and 7 with other lung neoplasms than NSCLCs) and 71 men (29 with adenocarcinoma, 27 with SqCC, 8 with other NSCLCs, and 7 with other lung neoplasms than NSCLCs). The majority of the patients were diagnosed at an early stage of lung cancer, with 43 (38.39%) diagnosed at stage I and 27 (24.11%) diagnosed at stage II.

We examined differences in biomarker concentrations across histopathological groups of lung cancer patients in terms of gender before comparing measured biomarkers across this groups. There were statistically significant differences in CRP concentration between female and male adenocarcinoma patients (*p* = 0.0411; meanF = 4.42 mg/L, meanM = 23.93 mg/L; raw mean difference [D] = 19.51 mg/L), MMP-2 concentration in SqCC patients (*p* = 0.0065; meanF = 114.10 ng/mL, meanM = 151.53 ng/mL; D = 37.43 ng/mL), and SAA_1_ concentration in other NSCLCs (*p* = 0.0068; meanF = 13.51 µg/mL, meanM = 298.14 µg/mL; D = 284.63 µg/mL). The only statistically significant difference between male and female in the analysis of the entire research group was the level of CRP (*p* = 0.0080; meanF = 10.24 mg/L, meanM = 29.58 mg/L; D = 19.34 mg/L). The average age (±standard deviation [SD]) of lung cancer patients was 67 ± 8, and no statistically significant differences between the groups were found. The average age of adenocarcinoma patients was 67 ± 8, that of SqCC patients 68 ± 8, that of other NSCLC patients 68 ± 12, and that of patients with other lung neoplasms than NSCLC was 66 ± 6.

### 3.2. Tumor Markers

**Figure 2:**
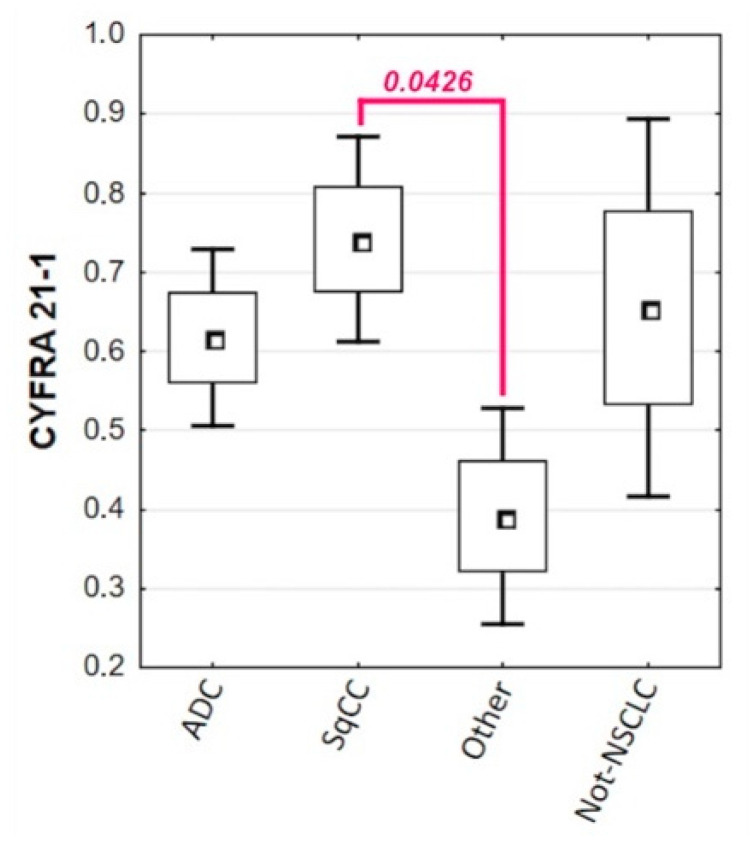
Distribution of CYFRA 21-1 levels in lung cancer subtypes with the statistically significant difference between patients with SqCC and other NSCLCs (*p* = 0.0426).

The only statistically significant difference in tumor marker concentrations in lung cancer patients was in the levels of CYFRA 21-1 between SqCC (me = 5.49 ng/mL) and other NSCLCs (me = 2.47 ng/mL; *p* = 0.0426). Figure 2 demonstrates the statistically significant distribution of CYFRA 21-1 concentrations in groups divided by lung cancer subtype. Table 2 compiles descriptive statistics for all tumor markers analyzed based on lung cancer type, subtype, and stage.

### 3.3. Inflammatory Markers

**Figure 3:**
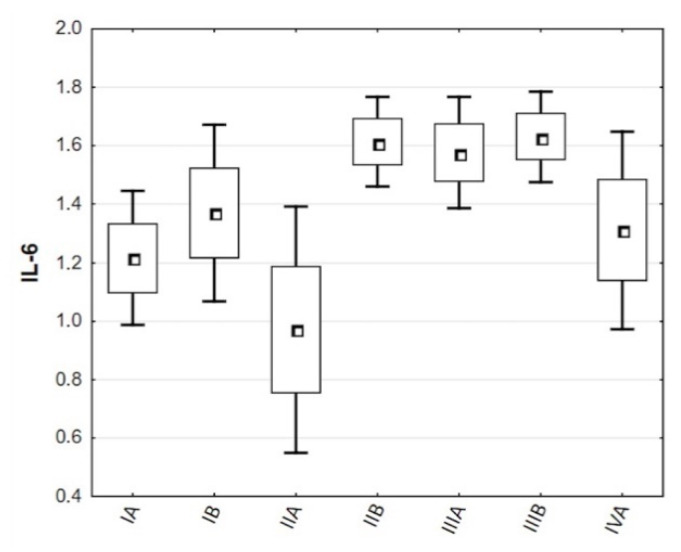
Distribution of IL-6 concentrations among lung cancer stages.

There were no statistically significant differences in any inflammatory marker between lung cancer types and subtypes. The concentrations of IL-6 between lung cancer stages were the only statistically significant difference in the analysis of variances (*p* = 0.0175). Tukey’s HSD analysis, on the other hand, did not reveal which groups significantly differ statistically. The graph displaying the distribution of IL-6 concentrations among groups indicates a peak in IL-6 concentrations in patients with stage IIB (me = 44.14 pg/mL), IIIA (me = 47.36 pg/mL), and IIIB (me = 37.00 pg/mL) lung cancer. Figure 3 depicts the distribution of IL-6 marker concentrations among lung cancer stages. Table 3 collects descriptive statistics of inflammatory markers according to lung cancer type, subtype, and stage.

### 3.4. Metabolic Markers

**Figure 4:**
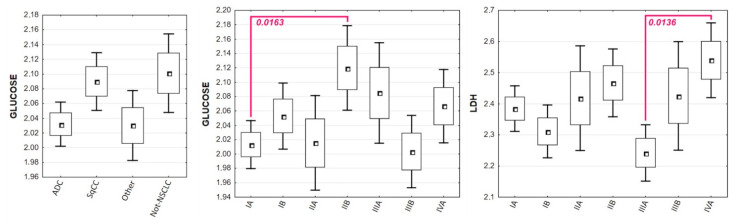
Distribution of glucose levels in lung cancer subtypes and stages, with a statistically significant difference between patients with stage IA and stage IIB lung cancer (*p* = 0.0163), and the activity of LDH between lung cancer stages, with a statistically significant difference between patients with stage IIIA and stage IVA lung cancer (*p* = 0.0136).

Glucose concentrations showed statistically significant differences both between lung cancer subtypes and stages. When compared to patients with adenocarcinoma (me = 100.0 mg/dL), SqCC (me = 119.0 mg/dL), and other NSCLCs (me = 105.0 mg/dL), patients with other lung neoplasms had the highest glucose values (me = 130.5 mg/dL). Furthermore, individuals with stage IA lung cancer had significantly lower glucose levels (me = 101.0 mg/dL) than patients with stage IIB lung cancer (me = 132.0 mg/dL; *p* = 0.0163). There were also statistically significant differences in LDH levels between lung cancer stages. Patients in stage IVA had considerably higher LDH activity (me = 317.07 U/L) than patients in stage IIIA (me = 159.60 U/L). Table 4 collects descriptive statistics of metabolic markers in relation to lung cancer type, subtype, and stage, whereas Figure 4 depicts statistically significant differences in glucose concentrations and LDH activity in lung cancer patients.

### 3.5. Correlation

**Figure 5:**
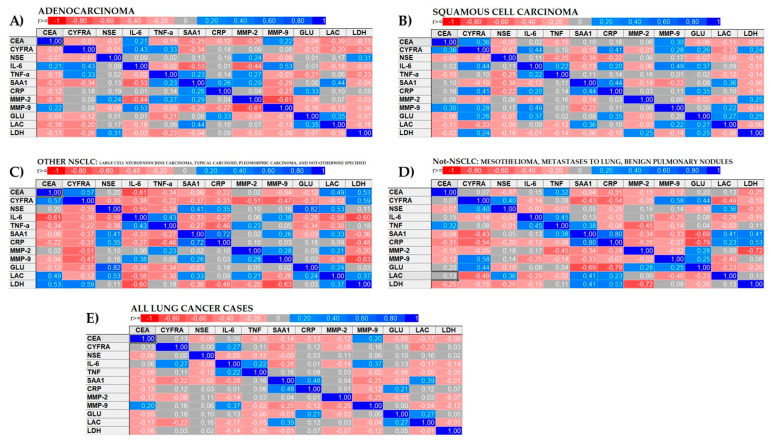
Heatmaps of the pairwise correlations between tumor, inflammatory, and metabolic marker concentrations in patients with (**A**) adenocarcinoma, (**B**) squamous cell carcinoma, (**C**) non-small cell lung carcinomas other than adenocarcinoma and squamous cell carcinoma, (**D**) lung neoplasms other than non-small cell lung carcinoma, and (**E**) in the entire research group without division into subgroups based on histopathological diagnosis. Correlation coefficients receive a range between −1 (red) and 1 (blue), showing a negative and positive relationship between biomarkers, respectively.

The pairwise correlation revealed a significant positive correlation between IL-6 and MMP-9 in adenocarcinoma and SqCC, with correlation coefficients of 0.53 and 0.49, respectively. There was also a significant positive correlation between acute-phase protein concentrations, SAA_1_, and CRP in patients with all types of lung neoplasm except adenocarcinoma, with correlation coefficients of 0.44 in SqCC, 0.72 in other NSCLC, and 0.80 in other lung neoplasms. Table 5 collects pairwise relationships, which are also graphically displayed as heatmaps in Figure 5.

### 3.6. Diagnostic Value of Biomarkers for Adenocarcinoma and Squamous Cell Carcinoma

Prior to examining the diagnostic value of individual and combined biomarkers, logistic regression analysis was used to analyze and exclude the potential effects of age, gender, and smoking status on biomarker concentrations. The only statistically significant difference between patients with adenocarcinoma and SqCC was in the levels of glucose. There were no differences in the statistical significance of the remaining biomarkers between adenocarcinoma and SqCC patients, both unadjusted and adjusted for age, gender, and smoking status. These results suggest that differences in the levels of these biomarkers in our case are not due to confounding variables such as age and gender. All calculations are collected and presented in the Table 6.

**Figure 6:**
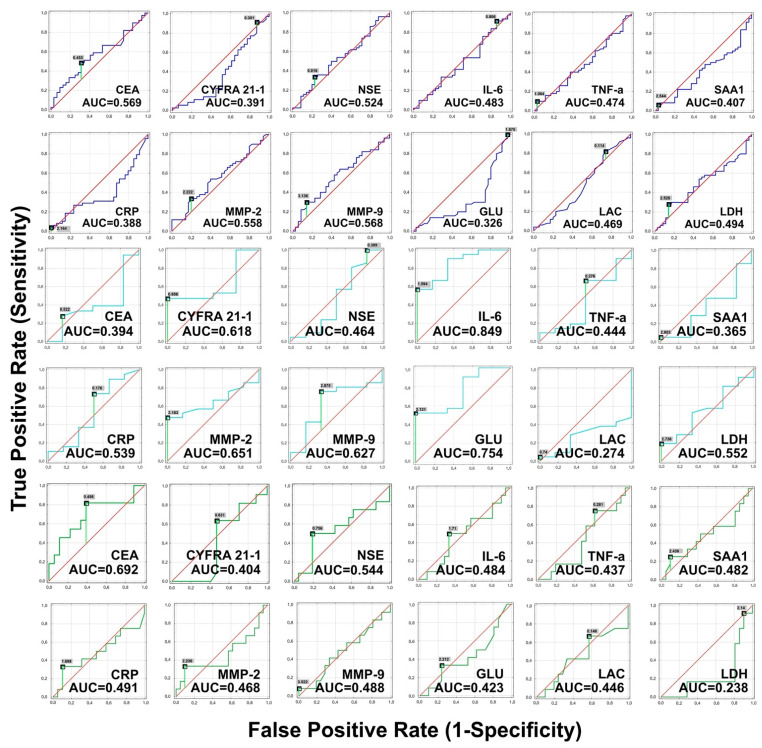
Receiver operating characteristic (ROC) curves evaluating the ability of single biomarkers to distinguish NSCLC patients with adenocarcinoma and squamous cell carcinoma (navy line), lung cancer stage IIB and IIA (teal line), and IIIA and IIB (green line).

Subsequently, ROC analysis with the calculation of AUC was performed to examine the ability of each of the 12 biomarkers to distinguish NSCLC patients with adenocarcinoma from patients with SqCC (results are collected in Table 7), and patients with lung cancer stages IIB and IIA (Table 8), and IIIA and IIB (Table 9). Figure 6 graphically presents the calculations of biomarkers diagnostic value in differential diagnoses.

CEA with AUC = 0.569, 48.7% sensitivity, and 68.8% specificity has the highest diagnostic values as single biomarker for distinguishing adenocarcinoma from SqCC patients. Glucose with AUC = 0.674, CRP with AUC = 0.612, and CYFRA 21-1 with AUC = 0.609 can identify SqCC from adenocarcinoma patients.

Lactate is the best single biomarker in the differential diagnosis of patients with stage IIA and IIB lung cancer, with AUC = 0.726, 100.0% sensitivity, and 52.4% specificity, whereas IL-6 with AUC = 0.849 may successfully distinguish patients with stage IIB from patients with stage IIA lung cancer.

LDH, on the other hand, is the best single biomarker in the differential diagnosis of stage IIB and IIIA lung cancer patients, with AUC = 0.762, 81.0% sensitivity, and 83.3% specificity. CEA (AUC = 0.692, 81.8% sensitivity, and 61.1% specificity) is the biomarker with the highest diagnostic value for distinguishing lung cancer stages IIIA and IIB.

The modeling of the combinations of markers into the panel of markers was the next step in the search for the best diagnostic tool for differential diagnosis of lung adenocarcinoma and SqCC, and lung cancer stages IIB and IIA, IIIA and IIB. For this aim, all biomarkers were mixed together, yielding thousands of combinations via GLM with the Akaike criterion included. The best model has the lowest AIC value and must fulfill two requirements: (1) the panel of biomarkers should be well-matched to the data, and (2) it should be as simple as feasible. This provided the best models, which comprised three or four biomarkers.

**Figure 7:**
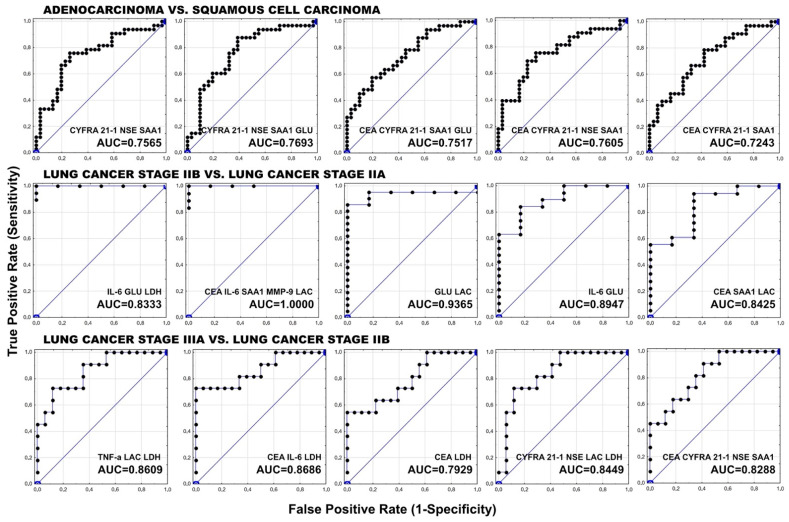
Receiver operating characteristic (ROC) curves evaluating the ability of multi-biomarker models to distinguish NSCLC patients with adenocarcinoma and squamous cell carcinoma, lung cancer stage IIB and IIA, and IIIA and IIB.

Table 10, Table 11 and Table 12 compile constructed models of biomarkers with the best AUC values for discriminating lung adenocarcinoma from SqCC (Table 10), stage IIB from IIA (Table 11), and stage IIIA from IIB (Table 12). Figure 7 uses ROC curves to graphically visualize these models.

When distinguishing lung adenocarcinoma and SqCC, the combination of CEA, CYFRA 21-1, NSE, and SAA_1_ has the highest sensitivity of 75.76%, a specificity of 70.97%, and an AUC value of 0.7605. The combination of these biomarkers with the exclusion of CEA results in a somewhat lower AUC value (0.7565) with the same sensitivity (75.76%) but slightly lower specificity (67.74%). The inclusion of glucose to this panel enhances the AUC value from 0.7565 to 0.7693 while lowering the sensitivity from 75.76% to 69.70% and maintaining the specificity at the same level (67.74%). Even though the model containing CYFRA 21-1, NSE, SAA_1_, and glucose has the highest AUC, the combination of CYFRA 21-1, NSE, and SAA_1_ has the lowest AIC.

The combinations of IL-6, glucose, and LDH, as well as CEA, IL-6, SAA_1_, MMP-9, and lactate, have 100% sensitivity and 100% specificity in distinguishing patients with stage IIB lung cancer from those with stage IIA, with the combination of IL-6, glucose, and LDH having a lower AUC value (0.8333 in comparison to 1.0000). The simple combination of glucose and lactate is also a useful diagnostic tool for distinguishing between lung cancer stages IIB and IIA, and is characterized by 95.24% sensitivity, 66.67% specificity, and an AUC value of 0.9365.

When distinguishing stage IIIA from IIB of lung cancer, the combination of CEA, IL-6, and LDH has the highest sensitivity of 72.73%, a specificity of 94.44%, and an AUC value of 0.8686, whereas a model with the lowest AIC value consists of TNF-α, lactate, and LDH, characterized by lower sensitivity (54.55%) and slightly lower specificity (88.24%) and AUC (0.8609).

## 4. Discussion

Lung cancer is a major problem in modern medicine. Despite numerous studies, our understanding of lung carcinogenesis and tumor progression remains unclear. Lung cancer is a multifactorial disease with a group of heterogeneous tumors, which complicates diagnostic and therapeutic approaches, as well as comprehension of the processes that underlie its pathogenesis. Current research efforts are specifically interested in the examination of changes in the TME at the molecular level [1,3,14]. In this retrospective study, we focused on nine biomarkers related to the processes occurring in the tumor microenvironment. We examined serum levels of pro- and anti-inflammatory cytokines (IL-6, TNF-α), which regulate immune responses, cell proliferation, and differentiation; matrix metalloproteinases (MMP-2, MMP-9), which remodel and degrade ECM and mediate cell–cell adhesion; acute-phase proteins (CRP, SAA_1_), which are linked to the expression of pro- and anti-inflammatory cytokines and play a role, among others, in the recruitment of immune cells to inflammatory sites; and metabolic markers (glucose, lactate, and LDH), which are associated with glucose metabolism in cancer cells, known as the Warburg effect. It is worth noting that our study group is representative and reflects the features of lung cancer patients as a whole: (1) NSCLC was the most often diagnosed type of lung cancer, (2) with an adenocarcinoma subtype predominating over SqCC, (3) women were mostly diagnosed with adenocarcinoma rather than SqCC, (4) and patients have been diagnosed in their sixth decade of life [8,9,20,21,42].

Chen et al. [43], in their work, referred to the “seed and soil” hypothesis by Stephen Paget (1889) in relation to the tumor microenvironment. The presence of tumor cells affects processes and changes in the TME, which in turn can affect tumor progression, invasion, and metastasis. Furthermore, changes in the TME might cause a systemic response of the host, manifested, for example, by the development of chronic inflammation, as seen by increased levels of acute-phase proteins, CRP, and SAA_1_. In another study, Mansuet-Lupo et al. [15] discovered that neutrophil and macrophage densities are related to oncogenic driver genes, varying among lung cancer subtypes. They discovered a high density of neutrophils and macrophages in tumors with wild-type epidermal growth factor receptor (EGFR) and a high density of neutrophils in tumors with b-raf proto-oncogene (BRAF) mutation, which affects the ability of tumor cell mutational status to change the pattern of cytokines and chemokines released by tumor cells and, thus, influences the TME’s chemoattractant properties. As a result, differences in the mutational status of lung cancer subtypes were expected to cause changes in the levels of cytokines IL-6 and TNF-α.

As normal tissue turns cancerous, matrix metalloproteinases are activated, and pro- and anti-inflammatory cytokines are excreted, resulting in extracellular matrix degradation and regulation of pathways activating inflammatory processes as well as pathways regulating proliferation, apoptosis, survival, and invasion. One example is IL-6’s involvement in the activation of the Janus kinases-signal transducer and activator of transcription proteins (JAK-STAT), mitogen-activated protein kinase (MAPK), and phosphoinositide 3-kinase/protein kinase B/mechanistic target of rapamycin (PI3K/AKT/mTOR) pathways [1]. Increasing cytokine and matrix metalloproteinase levels in the TME promote carcinogenesis and malignant progression, promoting subsequent microenvironmental changes. These markers are released into the blood at some level, allowing them to be measured in serum samples from patients. Although, no statistically significant changes in IL-6 and TNF-α levels are found between any group of lung cancer patients (adenocarcinoma, SqCC, other NSCLC, and other neoplasms than NSCLC), there are slightly higher levels of IL-6 in SqCC patients. Although IL-6 and TNF-α concentrations were not significantly different between our groups, their involvement in tumor growth should not be overlooked. Their function is shown by increased levels of acute-phase proteins and metabolic markers, which may be successfully measured in patients’ sera.

Particularly intriguing is the correlation between IL-6 and MMP-9 in patients with adenocarcinoma and SqCC, with correlation coefficients of 0.53, 0.49, respectively, which undoubtedly influences TME processes. Nie et al. [2] found that IL-6 serum concentrations of ≥4 pg/mL are associated with significantly poorer survival in both Americans and Caucasians with lung cancer. According to studies, IL-6 levels are higher in NSCLC patients compared to healthy controls, as well as in patients with metastatic NSCLC compared to undisseminated disease. Moreover, if IL-6 concentrations of ≥4 pg/mL are associated with poorer survival in lung cancer patients, and median values of this cytokine in patients with adenocarcinoma, SqCC, other NSCLC, and other lung neoplasms than NSCLC are, respectively, 34.74 pg/mL, 38.45 pg/mL, 31.43 pg/mL, and 25.63 pg/mL, we can conclude that IL-6 concentrations in our study group are elevated, and our patients are burdened with poorer outcomes. MMP-9, similar to IL-6, is produced by various tumor cells and inflammatory cells—neutrophils, eosinophils, monocytes, lymphocytes, and alveolar macrophages [44]. Animal studies showed that MMP-9 overexpression contributes to cancer development and progression. For example, cancer cells were less capable of colonizing the lung of MMP-9-deficient mice than the lung of wild-type mice, and MMP-9 null mice develop fewer cancers than wild-type mice [45]. Studies documented that MMP-9 was also involved in other steps of cancer development, including decreasing cancer cell apoptotic potential, promoting angiogenesis, and regulating immune responses to cancer, by altering cellular signals and regulating cytokines, growth factors, and angiogenic factors via complex cell–cell and cell–matrix interactions in the microenvironment [45,46,47]. There are reports of significant positive correlation between matrix metalloproteinases expression and metastatic capacities of cancer cells. Elevated IL-6 levels above the reference threshold and a correlation between this cytokine and the metastatic biomarker MMP-9 indicate a higher risk of lung cancer aggressiveness. The correlation between that cytokine and enzyme is an intriguing point in the ongoing exploration of their role in lung cancer, but this time together rather than separately.

Our research on the correlation between biomarkers in accordance with lung cancer type and subtype confirms the concept of tumor microenvironment that varies between types and subtypes. Moreover, evaluation of specific inflammatory and metabolic markers involved in TME alterations is the starting step for considering their diagnostic usefulness. The correlation of IL-6 and MMP-9, as well as other correlations in adenocarcinoma and SqCC and differences in the biomarker concentrations among groups, allowed us to observe subtype characteristics that might possibly be used to differentiate these two most common subtypes of lung cancer.

We looked at the relationship between well-known tumor markers and pro- and anti-inflammatory cytokines, matrix metalloproteinases, acute-phase proteins, and the Warburg effects’ markers, as well as their ability to distinguish adenocarcinoma from SqCC. Before modeling a biomarker panel to identify adenocarcinoma from SqCC, we evaluated each subtype’s patients’ age, gender, smoking status, and lung cancer stage as potential confounding factors that could interact with and influence biomarker usage. The best markers for differentiating adenocarcinoma from SqCC are glucose (AUC = 0.674), CRP (AUC = 0.612), and CYFRA 21-1 (AUC = 0.609), and there are no significant variations in their utility when corrected for age and gender.

All of the single biomarkers exhibit poor diagnostic sensitivity, specificity, and AUC in distinguishing adenocarcinoma from SqCC. We investigated a number of patterns and biomarker combinations that might efficiently, with the best sensitivity and specificity, diagnose adenocarcinoma and SqCC patients. However, none of the panels have an AUC greater than 0.80. The combination of CEA, CYFRA 21-1, NSE, and SAA_1_ has the highest diagnostic value among all available combinations (75.76% sensitivity, 70.97% specificity, and an AUC = 0.7605). In this panel, higher concentrations of CEA and NSE in adenocarcinoma and CYFRA 21-1 in SqCC are seen, which have histological explanations and overlap with the general picture of these lung cancer subtypes. The inclusion of SAA1 in a panel of markers distinguishing adenocarcinoma from SqCC patients is explained not only by histological, cellular, and molecular factors, but also by tumor localization in the lungs. Adenocarcinoma usually originates peripherally in the lungs, as opposed to SqCC, which grows centrally in the lung, where inflammation and irritation, particularly from cigarette smoke, may exert different biological characteristics than in the peripheral part of the lung. It causes differences in the levels of inflammatory markers between adenocarcinoma and SqCC [48,49]. Increased IL-6 and TNF-α concentrations in the TME result in the overproduction of acute-phase proteins, CRP, and SAA1. Despite the confirmation of these biomarkers’ involvement in lung cancer pathogenesis, we cannot consider the combination of CEA, CYFRA 21-1, NSE, and SAA1 as a panel that would successfully identify lung cancer subtypes and hence be included in the clinical diagnostic scheme of lung cancer patients. Combining biomarkers slightly improved the diagnostic characteristics of models distinguishing adenocarcinoma from SqCC, but the results are still unsatisfactory.

Knowing that changes in the TME, that is, remodeling of the extracellular matrix (ECM), pro- and anti-inflammatory processes, and cell metabolism, are affected by neoplastic progression, we conducted to examine the diagnostic potential of the studied biomarkers in relation to lung cancer stage. A peak in IL-6 concentrations in patients with stage IIB, IIIA, and IIIB lung cancer, with values of 44.14 pg/mL, 47.36 pg/mL, and 37.00 pg/mL, respectively, was one of the most notable changes in biomarker concentrations in relation to lung cancer stage. We also observed a characteristic increase in metabolic processes in advanced lung cancer stages, as evidenced by greater lactate concentrations and LDH activity in stages III and IV, as well as lower glucose levels due to increased glucose uptake. These alterations suggested that it could be useful in the differential diagnosis of certain lung cancer stages.

We determined that constructing a simple, easy, and sufficient tool capable of successfully differentiating patients with stage IIB and IIA lung cancer, as well as stage IIIA and IIB, would be clinically significant. Our choices are motivated by the first appearance of metastases (to lymph nodes) at stage IIB, as well as differences in treatment options between stages II and III, owing to the higher heterogeneity of stage III and the lack of a single therapy recommendation for all patients [50,51].

We found two combinations that can distinguish lung cancer patients with stage IIB from those with IIA with 100% sensitivity and 100% specificity, wherein IL-6, glucose, and LDH has an AUC = 0.8333 and the combination of CEA, IL-6, SAA1, MMP-9, and lactate an AUC = 1.0000. The combination of CEA, IL-6, and LDH, on the other hand, may distinguish patients with stage IIIA and stage IIB lung cancer with high sensitivity and diagnostic specificity. Increased levels of pro- and anti-inflammatory markers, as well as higher production of matrix metalloproteinases, are correlated with metastatic capacities of cancer cells, as seen by higher levels of these markers in more advanced stages of lung cancer.

In our study, we focused on the correlation of biomarkers involved in TME changes caused by ECM remodeling, pro- and anti-inflammatory processes, and cell metabolism, and we discovered some interrelationships that may influence lung cancer pathogenesis and malignant progression in relation to lung cancer subtype and stage. We also found a link between cytokines, matrix metalloproteinases, acute-phase proteins, Warburg effect markers, and tumor markers that have been proved to be clinically relevant in the detection of lung cancer. As a consequence, we discovered a few panels of markers that could separate lung adenocarcinoma from SqCC, as well as a few panels that could successfully differentiate patients with stages IIB and IIA as well as IIIA and IIB lung cancer.

We should consider the role of CEA, IL-6, SAA_1_, MMP-9, LAC, and LDH in the perspective of having readily accessible and inexpensive diagnostic tools that can be employed effectively in the differential diagnosis of lung cancer stages IIB and IIA, and IIIA and IIB, and improve therapeutic decision making. An undeniable advantage of such panels as the combinations of CEA, IL-6, SAA_1_, MMP-9, and lactate, IL-6, glucose, and LDH, or CEA, IL-6, and LDH is the ability of its detection in patients’ blood and use in a large group of NSCLC patients whose tumor tissue is not easily accessible, particularly in peripherally situated adenocarcinomas. Our panels of markers could be integrated into the scheme of lung cancer diagnostic procedures as a low-cost, simple, and practical diagnostic tools. In the future, performing multidimensional assessment of known markers may improve diagnostic and prognostic algorithms.

## Figures and Tables

**Table 1:** Patients’ characteristics according to the type of lung cancer.

	Non-Small Cell Lung Carcinoma			Non-NSCLC	Overall
	Adenocarcinoma	Squamous Cell Carcinoma (SqCC)	Other NSCLCs’ Subtypes		
N (%)	50 (44.64%)	35 (31.25%)	13 (11.61%)	14 (12.50%)	112 (100%)
Age					
Mean ± SD	67 ± 8	68 ± 8	68 ± 12	65 ± 5	67 ± 8
Range	39–81	47–82	40–84	54–71	39–84
Median	69	69	73	67	68
Gender					
Male	29 (58.00%)	27 (77.14%)	8 (61.54%)	7 (50.00%)	71 (63.39%)
Female	21 (42.00%)	8 (22.86%)	5 (38.46%)	7 (50.00%)	41 (36.61%)
Surgery					
Lobectomy	30 (60.00%)	19 (54.29%)	7 (53.85%)	5 (35.71%)	61 (54.46%)
Wedge resection	9 (18.00%)	9 (25.71%)	4 (30.77%)	6 (42.86%)	28 (25.00%)
Biopsy	6 (12.00%)	-	-	3 (21.43%)	9 (8.04%)
Segmentectomy	5 (10.00%)	1 (2.86%)	1 (7.69%)	-	7 (6.25%)
Bilobectomy	-	4 (11.43%)	1 (7.69%)	-	5 (4.46%)
Pulmonectomy	-	2 (5.71%)	-	-	2 (1.79%)
Stage					
IA1	2 (4.00%)	3 (8.57%)	-	-	5 (4.46%)
IA2	6 (12.00%)	6 (17.14%)	3 (23.08%)	-	15 (13.39%)
IA3	3 (6.00%)	1 (2.86%)	1 (7.69%)	-	5 (4.46%)
IB	12 (24.00%)	4 (11.43%)	2 (15.38%)	-	18 (16.07%)
IIA	3 (6.00%)	1 (2.86%)	1 (7.69%)	1 (7.14%)	6 (5.36%)
IIB	7 (14.00%)	12 (34.29%)	2 (15.38%)	-	21 (18.75%)
IIIA	9 (18.00%)	3 (8.57%)	-	-	12 (10.71%)
IIIB	4 (8.00%)	3 (8.57%)	2 (15.38%)	2 (14.29%)	11 (9.82%)
IVA	4 (8.00%)	2 (5.71%)	2 (15.38%)	6 (42.86%)	14 (12.50%)
IVB	-	-	-	-	-
Grading					
G1	1	-	-	-	1
G2	19	25	1	-	45
G3	19	-	1	-	20
NA	11	10	11	14	46
Smoking history					
Current	15 (30.00%)	10 (28.57%)	3 (23.08%)	3 (21.43%)	31 (27.68%)
Former	23 (46.00%)	21 (60.00%)	6 (46.15%)	5 (35.71%)	55 (49.11%)
Passive	1 (2.00%)	-	-	1 (7.14%)	2 (1.79%)
Never	2 (4.00%)	-	-	-	2 (1.79%)
NA	9 (18.00%)	4 (11.43%)	4 (30.77%)	5 (35.71%)	22 (19.64%)
Pack-years					
Mean ± SD	30 ± 17	36 ± 20	40 ± 26	38 ± 24	34 ± 19
Range	0–60	3–106	20–90	0–68	0–106
Median	30	38	35	36	30

SD—standard deviation; NA—not available; G1—grade 1, well differentiated; G2—grade 2, moderately differentiated; G3—grade 3, poorly differentiated.

**Table 2:** Levels of the tumor markers according to lung cancer type, subtype, and stage.

	Non-Small Cell Lung Carcinoma					Non-NSCLC	Overall
	ADC		SqCC	Other			
CEA [ng/mL]							
Mean ± SEM	4.72 ± 1.04		3.43 ± 0.76	2.80 ± 0.31		3.97 ± 1.65	3.96 ± 0.54
Range	1.26–36.08		1.14–26.28	1.83–4.65		1.78–20.38	1.14–36.08
Median	2.81		2.41	2.29		2.22	2.41
CYFRA 21-1 [ng/mL]							
Mean ± SEM	6.32 ± 1.77		7.63 ± 1,14	2.78 ± 0.40		7.20 ± 2.59	6.46 ± 0.88
Range	1.15–63.89		1.17–27.94	1.22–4.65		0.72–33.60	0.72–63.89
Median	3.89		5.49	2.47		4.27	4.13
NSE [ng/mL]							
Mean ± SEM	8.51 ± 2.07		7.38 ± 1.58	11.92 ± 5.13		7.03 ± 1.20	8.37 ± 1.21
Range	1.60–99.94		2.18–38.36	0.98–64.63		2.83–17.13	0.98–99.94
Median	4.45		4.24	4.37		5.31	4.42
	**IA**	**IB**	**IIA**	**IIB**	**IIIA**	**IIIB**	**IVA**
CEA [ng/mL]							
Mean ± SEM	2.71 ± 0.24	2.96 ± 0.55	6.36 ± 3.60	3.07 ± 0.36	6.92 ± 2.99	4.48 ± 2.00	5.07 ± 2.39
Range	1.26–5.95	1.88–10.21	1.83–24.35	1.81–8.31	1.87–36.08	1.78–20.38	1.14–26.28
Median	2.35	2.37	2.94	2.48	3.37	2.29	2.21
CYFRA 21-1 [ng/mL]							
Mean ± SEM	4.50 ± 0.77	3.28 ± 0.67	4.57 ± 1.33	7.86 ± 1.58	5.00 ± 0.70	5.43 ± 1.31	15.59 ± 5.75
Range	1.17–13.22	1.15–10.21	1.44–7.85	1.73–27.94	1.27–9.03	2.38–13.49	0.72–63.89
Median	3.2	2.23	4.49	4.13	4.53	4.23	11.7
NSE [ng/mL]							
Mean ± SEM	10.03 ± 2.97	11,48 ± 5.45	6.50 ± 2.30	4.91 ± 0.75	5.06 ± 0.77	8.35 ± 2.98	10.29 ± 2.80
Range	0.98–64.63	2.25–99.94	2.33–17.13	2.45–17.21	1.60–10.66	1.87–37.26	2.18–35.79
Median	4.44	3.93	4.22	3.51	4.93	5.37	5.74

SEM—standard error of the mean.

**Table 3:** Levels of the inflammatory markers according to lung cancer type, subtype, and stage.

	Non-Small Cell Lung Carcinoma				Non-NSCLC		Overall
	ADC	SqCC	Other				
IL-6 [pg/mL]							
Mean ± SEM	41.79 ± 5.39	42.04 ± 5.61	42.34 ± 12.13		33.65 ± 8.82		40.92 ± 3.43
Range	0.21–215.95	1.50–141.29	0.32–131.09		0.37–126.81		0.21–215.95
Median	34.74	38.45	31.43		25.63		34.34
TNF-α [pg/mL]							
Mean ± SEM	4.09 ± 0.82	15.47 ± 12.40	13.95 ± 8.55		27.97 ± 21.28		11.77 ± 4.79
Range	0.57–33.16	0.67–436.81	1.20–111.55		1.44–302.38		0.57–436.81
Median	2.13	2.33	2.63		2.83		2.37
SAA1 [µg/mL]							
Mean ± SEM	94.45 ± 17.74	119.66 ± 24.43	203.26 ± 61.77		82.01 ± 31.02		112.59 ± 13.70
Range	0.03–515.75	0.32–635.87	0.21–636.77		1.26–395.53		0.03–636.77
Median	12.16	83.17	208.23		32.62		45.63
CRP [mg/L]							
Mean ± SEM	21.65 ± 6.17	22.43 ± 5.28	29.54 ± 11.62		27.25 ± 12.40		23.39 ± 3.77
Range	0.22–217.67	0.40–109.66	0.60–114.20		0.60–153.93		0.22–217.67
Median	2.15	8.16	13.7		3.8		4.38
MMP-2 [ng/mL]							
Mean ± SEM	151.15 ± 5.08	142.97 ± 5.24	165.10 ± 19.10		152.64 ± 12.71		150.40 ± 3.88
Range	90.70–237.57	81.92–209.82	82.89–364.63		59.16–231.50		59.16–364.63
Median	144.09	141.97	158.76		144.39		143.95
MMP-9 [ng/mL]							
Mean ± SEM	1174.24 ± 97.77	1020.80 ± 110.68	916.97 ± 143.82		1190.65 ± 178.72		1098.48 ± 62.19
Range	73.03–2753.98	295.32–3327.25	104.94–1870.75		413.85–2371.49		73.03–3327.25
Median	1026.56	820.38	899.82		995.46		968.05
	**IA**	**IB**	**IIA**	**IIB**	**IIIA**	**IIIB**	**IVA**
IL-6 [pg/mL]							
Mean ± SEM	32.68 ± 8.91	40.47 ± 7.30	15.72 ± 6.29	54.42 ± 8.68	47.56 ± 8.86	50.37 ± 9.04	37.80 ± 9.36
Range	0.32–215.95	0.21–112.70	1.50–36.19	6.38–141.29	7.40–120.61	18.77–100.23	0.36–126.81
Median	21.1	39.37	8.52	44.14	47.36	37	28.86
TNF-α [pg/mL]							
Mean ± SEM	4.21 ± 1.00	5.31 ± 1.84	3.73 ± 1.02	24.85 ± 20.66	2.44 ± 0.37	17.18 ± 10.05	24.72 ± 21.37
Range	0.57–21.40	1.26–33.16	1.36–7.61	1.21–436.81	1.30–5.84	0.67–111.55	1.20–302.38
Median	2.1	2.68	2.95	2.33	2.18	2.2	2.79
SAA_1_ [µg/mL]							
Mean ± SEM	90.66 ± 22.79	133.35 ± 37.96	131.89 ± 61.54	95.38 ± 33.32	95.07 ± 36.25	98.03 ± 42.02	199.94 ± 48.35
Range	0.03–386.11	0.08–636.77	1.32–336.87	0.50–635.87	0.59–309.17	5.79–395.53	3.25–548.31
Median	35.56	101.97	75.59	7.58	10.09	13.3	211.35
CRP [mg/L]							
Mean ± SEM	12.94 ± 5.44	21.36 ± 9.16	20.15 ± 11.38	17.11 ± 6.93	22.77 ± 9.02	33.84 ± 13.96	50.94 ± 16.92
Range	0.60–93.34	0.25–114.20	0.60–64.00	0.60–109.66	0.22–81.32	1.08–153.93	0.60–217.67
Median	2.44	2.4	4.55	2.84	2.8	21.02	24.52
MMP-2 [ng/mL]							
Mean ± SEM	148.52 ± 7.97	161.15 ± 14.29	131.11 ± 5.22	143.06 ± 6.32	142.62 ± 11.87	140.81 ± 12.38	162.71 ± 8.96
Range	81.92–237.57	101.88–364.63	113.46–145.31	90.70–209.82	94.93–224.22	59.16–200.94	118.06–227.51
Median	152.7	142.99	133.87	143.99	132.16	144.27	153.14
MMP-9 [ng/mL]							
Mean ± SEM	996.67 ± 148.87	1103.62 ± 143.04	960.91 ± 276.48	1178.16 ± 145.49	1191.19 ± 231.05	1074.92 ± 149.41	1055.56 ± 139.83
Range	73.03–2753.98	396.52–2594.84	508.99–2279.45	387.99–2712.13	285.38–3327.25	647.48–2367.12	463.48–2283.19
Median	734.26	1018.32	660.99	973.91	1033.8	899.82	995.19

SEM—standard error of the mean.

**Table 4:** Levels of the metabolic markers according to lung cancer type, subtype, and stage.

	Non-Small Cell Lung Carcinoma				Non-NSCLC		Overall
	ADC	SqCC		Other			
Glucose [mg/dL]							
Mean ± SEM	111.42 ± 4.84	127.80 ± 6.48		109.38 ± 6.91	129.43 ± 7.94		118.55 ± 3.29
Range	75.00–251.00	70.00–268.00		83.00–180.00	82.00–184.00		70.00–268.00
Median	100	119		105	130.5		105.5
Lactate [mmol/L]							
Mean ± SEM	2.17 ± 0.16	2.25 ± 0.18		2.73 ± 0.75	2.44 ± 0.34		2.29 ± 0.13
Range	0.60–6.90	0.80–5.00		0.50–9.70	1.30–5.70		0.50–9.70
Median	2.05	2.2		1.8	1.95		2
LDH [U/L]							
Mean ± SEM	288.24 ± 31.26	280.66 ± 36.06		311.43 ± 47.53	316.08 ± 51.09		292.04 ± 19.82
Range	97.80–1217.00	111.86–1217.50		113.28–777.48	131.40–824.56		97.80–1217.50
Median	213.06	205.57		261.55	250.08		218.67
	**IA**	**IB**	**IIA**	**IIB**	**IIIA**	**IIIB**	**IVA**
Glucose [mg/dL]							
Mean ± SEM	105.08 ± 4.36	116.06 ± 7.10	105.17 ± 8.06	138.76 ± 10.83	126.50 ± 11.21	102.64 ± 6.46	119.50 ± 7.65
Range	70.00–163.00	90.00–197.00	83.00–127.00	89.00–268.00	91.00–209.00	82.00–145.00	89.00–180.00
Median	101	103.5	104	132	105.5	94	110
Lactate [mmol/L]							
Mean ± SEM	2.36 ± 0.36	2.38 ± 0.18	2.92 ± 0.53	2.08 ± 0.26	1.79 ± 0.29	1.82 ± 0.31	3.04 ± 0.50
Range	0.80–9.70	1.20–3.70	1.80–5.00	1.00–5.50	0.60–4.00	0.50–4.10	1.10–6.90
Median	1.9	2.3	2.25	1.6	1.4	1.8	2.7
LDH [U/L]							
Mean ± SEM	274.49 ± 41.33	226.19 ± 28.61	288.31 ± 56.60	351.66 ± 55.88	187.11 ± 23.35	332.96 ± 74.99	401.87 ± 71.10
Range	136.25–1217.00	113.28–558.59	144.61–513.24	116.77–1217.50	97.80–360.97	111.86–824.56	174.03–1149.40
Median	223.92	188.43	266.85	277.85	159.6	205.57	317.07

SEM—standard error of the mean.

**Table 5:** The pairwise correlation of tumor, inflammatory, and metabolic marker combinations among the lung cancer groups studied.

Marker’s Combination	Non-Small Cell Lung Carcinoma			Non-NSCLC
	ADC	SqCC	Other	
CEA + CYFRA 21-1	NS	*p* = 0.048; r = 0.36	NS	NS
CEA + LAC	*p* = 0.024; r = −0.39	NS	NS	NS
CYFRA 21-1 + IL-6	*p* = 0.014; r = 0.43	*p* = 0.012; r = 0.44	NS	NS
CYFRA 21-1 + CRP	NS	*p* = 0.023; r = 0.41	NS	NS
NSE + SAA_1_	NS	*p* = 0.034; r = −0.38	NS	NS
NSE + GLU	NS	NS	*p* = 0.007; r = 0.82	NS
IL-6 + SAA_1_	*p* = 0.002; r = −0.53	NS	NS	NS
IL-6 + MMP-2	*p* = 0.010; r = −0.44	NS	NS	NS
IL-6 + MMP-9	*p* = 0.002; r = 0.53	*p* = 0.006; r = 0.49	NS	NS
IL-6 + GLU	NS	*p* = 0.041; r = 0.37	NS	NS
SAA_1_ + CRP	NS	*p* = 0.014; r = 0.44	*p* = 0.028; r = 0.72	*p* = 0.003; r = 0.80
SAA_1_ + GLU	NS	NS	NS	*p* = 0.019; r = −0.69
SAA_1_ + LAC	*p* = 0.011; r = 0.44	*p* = 0.045; r = 0.36	NS	NS
CRP + GLU	NS	NS	NS	*p* = 0.004; r = −0.79
MMP-2 + MMP-9	*p* = 0.000; r = −0.61	NS	NS	NS
MMP-2 + LDH	NS	NS	NS	*p* = 0.012; r = −0.72
GLU + LAC	*p* = 0.045; r = 0.35	NS	NS	NS

NS—not significant.

**Table 6:** Logistic regression analyses on the influence of confounding variables on biomarkers in distinguishing adenocarcinoma from squamous cell carcinoma patients.

	Unadjusted			Adjusted by Age, Gender, and Smoking Status		
Biomarker	OR	95% CI	*p*-Value	OR	95% CI	*p*-Value
CEA	1.28	−0.75–3.20	0.22	1.75	−0.37–6.08	0.08
CYFRA 21-1	2.78	−2.47–0.41	0.16	1.93	−1.61–1.62	0.99
NSE	∞	−1.08–1.60	0.70	1.56	−1.64–1.69	0.98
IL-6	∞	−1.04–0.77	0.77	1.75	−1.13–0.86	0.79
TNF-α	∞	−1.32–0.85	0.68	2.25	−1.56–0.93	0.62
SAA_1_	0.71	−0.73–0.14	0.18	0.99	−0.83–0.15	0.17
CRP	1.33	−1.04–0.09	0.10	2.55	−0.78–0.60	0.80
MMP-2	1.46	−2.11–6.77	0.30	2.02	−0.65–10.84	0.08
MMP-9	0.00	−0.95–2.22	0.43	1.75	−0.97–3.08	0.31
GLU	2.82	−8.61–−0.52	0.03	5.18	−8.93–0.18	0.06
LAC	∞	−2.45–1.71	0.72	1.72	−3.47–1.52	0.44
LDH	∞	−1.84–1.91	0.97	2.57	−1.65–2.44	0.70

CI—confidence interval; NA—not available; OR—odds ratio.

**Table 7:** The diagnostic efficiency of single biomarkers to distinguish NSCLC patients with adenocarcinoma and squamous cell carcinoma.

Biomarker	*p*-Value	ADC vs. SqCC			SqCC vs. ADC		
		Sensitivity	Specificity	AUC	Sensitivity	Specificity	AUC
CEA	0.3131	48.7%	68.8%	0.569	71.9%	33.3%	0.431
CYFRA 21-1	0.1256	91.4%	12.9%	0.391	45.2%	85.7%	0.609
NSE	0.7077	34.0%	77.1%	0.524	8.6%	98.0%	0.476
IL-6	0.7968	92.0%	14.3%	0.483	68.6%	46.0%	0.517
TNF-α	0.6799	10.0%	97.1%	0.474	60.0%	50.0%	0.526
SAA1	0.1364	6.0%	97.1%	0.407	88.6%	34.0%	0.593
CRP	0.0762	4.2%	100.0%	0.388	64.7%	68.8%	0.612
MMP-2	0.3604	34.0%	80.0%	0.558	14.3%	88.0%	0.442
MMP-9	0.2822	30.0%	85.7%	0.568	88.6%	16.0%	0.432
GLU	0.0048	100.0%	2.9%	0.326	74.3%	70.0%	0.674
LAC	0.6378	82.0%	25.7%	0.469	42.9%	70.0%	0.531
LDH	0.9281	28.0%	85.7%	0.494	94.3%	20.0%	0.506

AUC—area under the curve.

**Table 8:** The diagnostic efficiency of single biomarkers to distinguish patients with stage IIB and IIA lung cancer.

Biomarker	*p*-Value	IIA vs. IIB			IIB vs. IIA		
		Sensitivity	Specificity	AUC	Sensitivity	Specificity	AUC
CEA	0.4412	83.3%	61.1%	0.606	27.8%	83.3%	0.394
CYFRA 21-1	0.4163	75.0%	47.1%	0.382	47.1%	100.0%	0.618
NSE	0.8207	50.0%	76.2%	0.536	100.0%	16.7%	0.464
IL-6	0.0000	∞	∞	0.151	57.1%	100.0%	0.849
TNF-α	0.6987	50.0%	81.0%	0.556	66.7%	50.0%	0.444
SAA1	0.3079	83.3%	52.4%	0.635	4.8%	100.0%	0.365
CRP	0.7968	33.3%	84.2%	0.461	73.7%	50.0%	0.539
MMP-2	0.1448	100.0%	14.3%	0.349	47.6%	100.0%	0.651
MMP-9	0.3411	100.0%	14.3%	0.373	85.7%	16.7%	0.627
GLU	0.0192	∞	∞	0.246	52.4%	100.0%	0.754
LAC	0.0223	100.0%	52.4%	0.726	4.8%	100.0%	0.274
LDH	0.6877	66.7%	42.9%	0.448	19.0%	100.0%	0.552

AUC—area under the curve.

**Table 9:** The diagnostic efficiency of single biomarkers to distinguish patients with stage IIIA and IIB lung cancer.

Biomarker	*p*-Value	IIB vs. IIIA			IIIA vs. IIB		
		Sensitivity	Specificity	AUC	Sensitivity	Specificity	AUC
CEA	0.0760	88.9%	18.2%	0.308	81.8%	61.1%	0.692
CYFRA 21-1	0.3837	41.2%	100.0%	0.596	63.6%	52.9%	0.404
NSE	0.6977	100.0%	16.7%	0.456	50.0%	81.0%	0.544
IL-6	0.8809	23.8%	91.7%	0.516	50.0%	66.7%	0.484
TNF-α	0.5366	47.6%	83.3%	0.563	75.0%	38.1%	0.437
SAA1	0.8276	81.0%	41.7%	0.518	25.0%	90.5%	0.482
CRP	0.9395	94.7%	25.0%	0.509	33.3%	89.5%	0.491
MMP-2	0.7835	57.1%	66.7%	0.532	33.3%	90.5%	0.468
MMP-9	0.9118	19.0%	91.7%	0.512	8.3%	100.0%	0.488
GLU	0.4663	71.4%	50.0%	0.577	33.3%	76.2%	0.423
LAC	0,6246	100.0%	25.0%	0.554	66.7%	42.9%	0.446
LDH	0.0036	81.0%	83.3%	0.762	91.7%	9.5%	0.238

AUC—area under the curve.

**Table 10:** The diagnostic efficiency of multi-biomarker models to distinguish NSCLC patients with adenocarcinoma from patients with squamous cell carcinoma.

Marker Combinations	AIC	*p*-Value	OR	log OR	Sensitivity	Specificity	AUC
CYFRA 21-1 + NSE + SAA_1_	82.96	0.0088	6.56	1.88	75.76%	67.74%	0.7565
CYFRA 21-1 + NSE + SAA_1_ + GLU	83.59	0.0046	4.83	1.57	69.70%	67.74%	0.7693
CEA + CYFRA 21-1 + SAA_1_ + GLU	84.25	0.0061	3.18	1.16	69.70%	58.06%	0.7517
CEA + CYFRA 21-1 + NSE + SAA_1_	84.55	0.0069	7.64	2.03	75.76%	70.97%	0.7605
CEA + CYFRA 21-1 + SAA_1_	85.09	0.0090	2.77	1.02	66.67%	58.06%	0.7243

AIC—Akaike information criterion; AUC—area under the curve; OR—odds ratio.

**Table 11:** The diagnostic efficiency of multi-biomarker models to distinguish stage IIB NSCLC patients from stage IIA NSCLC patients.

Marker Combinations	AIC	*p*-Value	OR	log OR	Sensitivity	Specificity	AUC
IL-6 + GLU + LDH	8.23	0.0000	∞	∞	100.00%	100.00%	0.8333
CEA + IL-6 + SAA_1_ + MMP-9 + LAC	12.00	0.0001	∞	∞	100.00%	100.00%	1.0000
GLU + LAC	21.95	0.0018	40.00	3.69	95.24%	66.67%	0.9365
IL-6 + GLU	22.14	0.0033	17.00	2.83	89.47%	66.67%	0.8947
CEA + SAA_1_ + LAC	26.90	0.0442	17.00	2.83	94.44%	50.00%	0.8425

AIC—Akaike information criterion; AUC—area under the curve; OR—odds ratio.

**Table 12:** The diagnostic efficiency of multi-biomarker models to distinguish stage IIIA NSCLC patients from stage IIB NSCLC patients.

Marker Combinations	AIC	*p*-Value	OR	log OR	Sensitivity	Specificity	AUC
TNF-α + LAC + LDH	31.75	0.0032	9.00	2.20	54.55%	88.24%	0.8609
CEA + IL-6 + LDH	34.14	0.0063	45.33	3.81	72.73%	94.44%	0.8686
CEA + LDH	34.49	0.0067	9.60	2.26	54.55%	88.89%	0.7929
CYFRA 21-1 + NSE + LAC + LDH	36.27	0.0057	9.00	2.20	54.54%	88.24%	0.8449
CEA + CYFRA 21-1 + NSE + SAA_1_	37.37	0.0380	8.17	2.10	63.64%	82.35%	0.8288

AIC—Akaike information criterion; AUC—area under the curve; OR—odds ratio.

## Data Availability

The data presented in this study are available on request from the corresponding author.

## Abbreviations

AD	adenocarcinoma
AIC	Akaike information criterion
AJCC	American Joint Committee on Cancer
ALK	anaplastic lymphoma kinase
AMMS	Asseco Medical Management Solutions
ANOVA	analysis of variance
AUC	area under the curve
BRAF	b-raf proto-oncogene
CEA	carcinoembryonic antigen
CI	confidential interval
CRP	C-reactive protein
CYFRA 21-1	cytokeratin 19
ECM	extracellular matrix
EDTA	ethylenediaminetetraacetic acid
EGFR	epidermal growth factor receptor
EIA	enzyme immunoassay
ELISA	enzyme-linked immunosorbent assay
GLM	generalized linear model
GLU	glucose
GLUT	glucose transporter
HRP	horseradish peroxidase
HSD	honestly significant difference (Tukey’s honestly significant difference test)
IL-6	interleukin-6
JAK-STAT	Janus kinases-signal transducer and activator of transcription proteins
LAC	lactate
LDH	lactate dehydrogenase
MAPK	mitogen-activated protein kinase
MCT	monocarboxylate transporter
MMP-2	matrix metalloproteinase-2
MMP-9	matrix metalloproteinase-9
NA	not available
NCCN	National Comprehensive Cancer Network
NK	natural killer
NN	not NSCLC/not non-small cell lung carcinoma
NS	not significant
NSCLC	non-small cell lung carcinoma
NSE	neuron-specific enolase
OR	odds ratio
Ot	other NSCLC/other non-small cell lung carcinoma
PI3K/AKT/mTOR	phosphoinositide 3-kinase/protein kinase B/mechanistic target of rapamycin
ROC	receiver operating characteristic
SAA_1_	serum amyloid A_1_
SCLC	small cell lung carcinoma
SD	standard deviation
SEM	standard error of the mean
SqCC	squamous cell carcinoma
TMB	tetramethylbenzidine
TME	tumor microenvironment
TNM	TNM Classification of Malignant Tumors (tumor—lymph node—metastasis)
TNF-α	tumor necrosis factor-α
VAT	video-assisted thoracoscopy
